# A Re-Interpretation of the ‘Two-child Norm’ in Post-Transitional Demographic Systems: Fertility Intentions in Taiwan

**DOI:** 10.1371/journal.pone.0135105

**Published:** 2015-08-20

**Authors:** Stuart Basten, Georgia Verropoulou

**Affiliations:** 1 Department of Social Policy and Intervention, University of Oxford, Oxford, United Kingdom; 2 Department of Statistics and Insurance Science, University of Piraeus, Piraeus, Greece; London School of Economics, UNITED KINGDOM

## Abstract

Taiwan currently has one of the lowest fertility rates in the world, leading to projections of rapid population ageing and decline. In common with other territories in Pacific Asia, policies designed to support childbearing have recently been introduced. Some optimism for the future success of these policies has been drawn from the fact that the ‘ideal’ number of children stated in Taiwanese surveys is over two. In this way, Taiwan appears to fit the ‘two-child norm’ model identified for Europe and North America. Furthermore, this feature has led commentators to state that Taiwan is not in a ‘low fertility trap’–where positive feedback mechanisms emanating from the normalisation of small families, slow economic growth and ageing/declining population mean attempts to increase fertility become ever less likely to succeed. Using a recent national representative survey, and arguing that ‘intentions’ are a more reliable guide to understanding the circumstances of family formation, this paper explores fertility intentions in Taiwan with a special focus on women at parity one and parity two. This will form the first full-length examination of fertility intentions in Taiwan published in English and one of the few studies of Pacific Asia that reports a micro-level analysis. We argue that using intentions should provide a better ‘barometer’ of attitudes towards childbearing in Taiwan, and that through micro-level analysis, we can better identify the predictors of intentions that could, in turn, provide useful clues both for projections as well as shaping policy responses. While we found some evidence for a ‘two-child norm’ among childless women, this could be an unrealistic ideal. This is supported by the fact that a majority of women with one child do not intend to have another.

## Introduction

**Fig 1:**
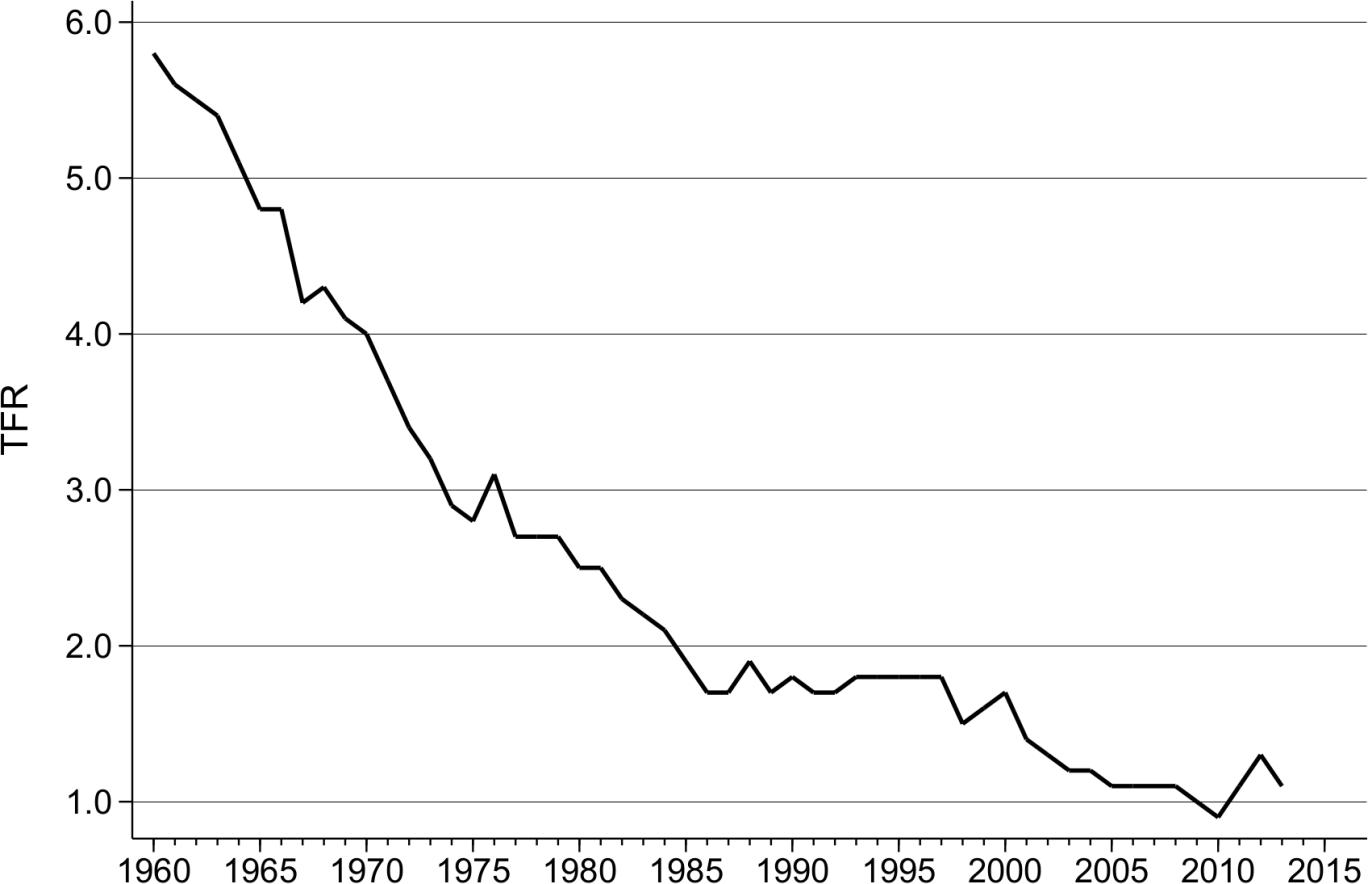
Total fertility rates in Taiwan, 1960–2014. Source: [1].

Taiwan has one of the lowest total fertility rates [TFR] in the world. As Fig 1 demonstrates, there has been a precipitous decline from a TFR of almost six children per woman in the early 1960s, to a nadir of 0.9 in 2010, while the TFR for 2014 was 1.06 [1]. In the two most populous cities–Taipei and Kaohsiung–the 2010 TFR was 0.89 and 0.84 respectively, while in the port city of Keelung, TFR in 2010 was just 0.73 [2]. Demographically, there has been a significant shift towards much later childbearing and the postponement (and indeed avoidance) of marriage [2]. While postponement of marriage and birth can certainly ‘artificially depress’ the TFR through the so-called ‘tempo effect’ [3], a recent influential study suggests that ‘recuperation’ of higher order births at older ages is less important for Taiwan [4]. This assumption of a genuine sinking to very low *quantum* fertility is confirmed in cohort measurements and forecasts. In the recent study by Myrskala et al. [5], the 1979 cohort fertility forecast for Taiwan is just 1.35, ranked 37 out of 37 developed countries. Furthermore, in contrast to the reversal in cohort fertility trends seen elsewhere, Taiwan, they estimate, ‘will continue to show notable declines in lifetime fertility’ (p.32).

Currently the total population aged 15–64 is 17.4 million, compared to 2.8 million aged over 65. Taiwanese government projections estimate that by 2060 these figures will be 9.2 million and 7.4 million respectively. In other words, the proportion of the population aged 65 and above is forecast to rise from 12.0% to 40.6% in 2060. Not only is the country forecast to age rapidly, but the overall population is forecast to shrink by 5.3 million (or 22.3%)–somewhat higher than the UN forecasts of 3.7 million [6], although the model used there has been critiqued elsewhere [7].

The twin challenges of population ageing and population decline have been discussed extensively in the academic literature (see [8] for a useful overview). In terms of welfare, population ageing poses challenges to the maintenance (and expansion) of the National Health Insurance system, which already ‘has difficulties in caring for older patients with multiple comorbidities, complex care needs, functional impairments, and post-acute care needs’ [9] (p.s23) as well as the pension system [10] in terms of sizes of both the tax base and the number potentially in receipt of benefits. Although this very pessimistic view of population ageing has been challenged in many ways (such as considering the release of savings from elderly people providing a ‘second demographic dividend’ [11] as well as taking into account improved healthy life expectancy in measuring ‘old age’ and ‘dependency’ [12]), the scope of change is certain beyond any doubt. Finally, while some have questioned the ‘moral panic’ over population decline [13], the economic and security implications for Taiwan are clear. Indeed, in 2011, Taiwanese President Ma Ying-jeou was quoted as saying that ‘the low birth rate is a serious national security threat’ (quoted in [14], p.22). Similarly, Hau Lung-Bin, the mayor of Taipei, stated in his inaugural address that ‘The falling birthrates have made a significant impact on our already graying population over the recent years…This phenomenon will indeed cripple our city’s development’ (quoted in [2], p.85).

In common with other Pacific Asian countries, effective anti-natalist (family planning) policies played an important role in fertility decline in Taiwan–cited by one study as ‘one of the first and most successful national family planning programs, at a time when successes were few’ [15] (p.99). While replacement fertility was reached in 1984, it was not until 2006 that specific pro-natalist measures were implemented in the form of the ‘Mega Warmth Programme’ (though pro-natalist *statements* were made in 1992)–again a turnaround common with other Pacific Asian countries [16]. In recent years a number of national measures have been designed to boost childbearing. These have been broadly based around seven ‘themes’: developing childcare facilities; financial assistance for families; creation of family-friendly workplaces; revision of maternity protection; improvement of the reproductive care system; creating child-safe environments and expanding the opportunities to meet prospective partners. They have been realised through various financial incentives such as childcare subsidies, ‘baby bonuses’, income tax rebates and housing subsidies, as well as free pre-school education and enhanced parental leave opportunities. These have been supplemented by local initiatives, most notably in Taipei [2]. There has long been a tension between ‘supporting the reconciliation of work and family’ and the feminist view that such policies ‘represent a sort of instrumentalization of women’s body, taking women’s body as an instrument to fulfil [a] nation’s target goal’ [17] (p.80).

The sociological and economic explanations for low fertility are covered in depth elsewhere for Taiwan [2,8,17–19] and for Pacific Asia more broadly [4,7,16,20]. Briefly, though, these reasons revolve around very high *direct* costs of childbearing associated with costs of living and (expected) investment in education and high *indirect* costs, especially for women, in the sphere of an ‘incomplete gender revolution’ [21–23] where changes in the *public* domain have not been met with changes in the *domestic*. Related to this, as Yu [24] observes, the changing nature of the Taiwanese labour market means that higher-educated younger Taiwanese women are forced to choose either their career or family in the formal labour market, while older generations were more able to combine family duties with *informal* sector work. Other issues such as working hours, limited welfare provision, the burden of caring for aged parents (and in-laws), the maintenance of hypergamous marriage systems (and the growth of cross-border marriages) are also important, as are some of the themes associated with the ‘second demographic transition’ [25] such as aspiring to greater levels of self-actualisation and more flexible life-course organisation.

While some scholars are generally optimistic about the future prospects of the twin roles of policy and future economic investment in increasing fertility [26], others are less so, citing the relative lack of success in other territories in the region where broadly pro-natalist policies have been implemented. Indeed, the Taiwanese government is forecasting only a modest rise in TFR to 1.10 by 2029, then stagnation to 2060 (in contrast to the UN–see [27]–although as noted this model has been criticised). As Frejka et al. [4] observe for the region as a whole, ‘public and private institutions are not devoting sufficient attention to generating broad social change supportive of parenting’ (p.579). In particular, they note that because ‘patriarchal customs and attitudes in the family, the workplace, and the political domain are deeply engrained…changing the patriarchal social environment will require special focus on policies to increase male involvement in the household and in the upbringing of women and to change the attitudes of employers’. They conclude that ‘unless current conditions are radically changed and child- and family-friendly environments are fostered, it is difficult to believe that fertility patterns will change’ (p.603).

Fertility preferences have been studied extensively within demography. It has been argued that fertility preferences can be a useful tool for (assisting in the) forecasting of fertility trends at the micro-level then extrapolating to the macro-level [28]. They can also be seen as a sort of ‘barometer’ for the ways that citizens feel a family ‘should’ look either on a macro- or micro-level. This is often measured through survey questions around ‘ideal family sizes’. In recent years, reported ‘ideal family sizes’ in the EU and other OECD countries have generally been higher than actual fertility rates (sometimes significantly so) [29]. While some have interpreted this gap as a reflection of respondents being ‘unrealistic about their fertility preferences or that ideals are too abstract and removed from real decision-making’ [30] (p.393), others have seen this as representing an important ‘space’ for fertility enhancing policies to work. For example, a European Commission Green Paper published in 2005 [31] interpreted the ‘gap’ between ideal and actual fertility as meaning that ‘if appropriate mechanisms existed to allow couples to have the number of children they want, the fertility rate could rise overall’ (p.5).

In this context, it is important to note the orthodox view regarding ideal number of children as reported in surveys from low-fertility Pacific Asia. Numerous studies observe that while fertility may have fallen to very low levels, ‘ideal family size’ has consistently remained at, or above, two children (for Singapore, see [32]; for Japan, see [33]; for Korea, see [34]). As in the example of the European Commission Green Paper [31] above, this ‘aspiration’ to have two children has been explicitly used by Pacific Asian governments as a bedrock of justifying extensive family policy instruments with a more or less explicitly pronatalist message (for Singapore, see [35], for Japan, see [36]). This has been the case in the limited number of studies that consider contemporary Taiwan [37] as well as ‘headline’ figures from recent ‘Knowledge, Attitude and Practice’ family planning surveys. (See [38–39] for earlier studies of fertility intentions in Taiwan under conditions of higher fertility.) This has led Sobotka and Beaujouan [30] in a recent important article to include Taiwan (along with Japan and South Korea) as an extension of their findings of a ‘two-child norm’ in Europe–based at least on ‘ideals’.

The significance of this is clear. Within the influential theoretical framework of the ‘low fertility trap’, it is posited that when a country sees ‘very low’ fertility for an extended period of time, a series of self-reinforcing mechanisms can mean that it becomes ever more challenging to increase fertility [40]. These mechanisms include a demographic one (i.e. a smaller number of births emanating from a smaller pool of reproductive age mothers) and an economic one (i.e. a variation of Easterlin’s [41,42] relative income hypothesis where (consumption) aspirations of the young are stymied by the effect of ageing on economic growth and intergenerational justice, which is then translated into lower fertility). The third, ‘sociological’, mechanism suggests that as the number of people with young children declines, this becomes reflected in declines in ‘personal ideal family size’. In later discussions of this mechanism, other authors suggest that this ‘socialisation of small family sizes’ can be further emphasised in the media and other outlets (e.g. advertising) [43–45]. As ideals fall, at least in the interpretations set out above, there is less ‘space’ for policies to work. As Peter MacDonald notes, ‘if these studies [of fertility ideals in Pacific Asia listed above] and their interpretations are correct, advanced Asian countries are not yet in the situation of the low-fertility trap where individual ideals fall to a level that corresponds with the number of children that people are actually having’ [46] (p.29).

There are, however, important ways in which this interpretation can be subjected to criticism. First, a number of studies have identified that sub-replacement ideals do actually exist in large populations such as China [47–50] as well as in parts of urban India [51,52] and Thailand [53]. Secondly, many of the surveys cited for Pacific Asia are based on relatively small samples (such as the World Values Survey) and, referring to a theme we refer to later, only include married women in their analysis. Given the large-scale ‘retreat from marriage’, ignoring the unmarried in any analysis of ‘ideals’ reveals only a very partial picture. A third critique revolves around the very notion of what an ‘ideal number of children’ really means. It has been argued, for example, that these ‘ideals’ are ‘clearly not well suited for predicting fertility levels’ [30] (p.393), given that such ideals might simply be a reflection of either social norms or idealised personal aspirations (which indeed may not be met). This might help explain the growing disparity between fertility ideals reported in surveys and actual fertility–especially in studies focussing on Pacific Asia.

This is not to say, however, that ‘ideal family size’ is, in and of itself, a ‘worthless measurement’. From a sociological and anthropological perspective, it is surely highly significant that a near universal two-child ideal has been identified in settings characterised by such different demographic as well as cultural and socioeconomic characteristics [30]. It is tempting, perhaps, to say that the notion of the ‘ideal family size’ might represent some kind of ‘potential fertility rate’ if the appropriate policies to meet aspirations were implemented–as anticipated in the European Commission’s Green Paper referred to earlier. However, while there have been large-scale policy interventions (explicitly or implicitly) designed with the intention of increasing the fertility rate across many settings in Pacific Asia, the impact upon the actual fertility rate (or the gap between the rate and the ideal family size) has been generally modest, thus insinuating a rather narrow use of ‘ideals’ in such a simple form.

There has been the general move towards a consideration of ‘intentions’ rather than necessarily focussing on ‘ideals’ [54–56]. Given that fertility (it can be argued) is ‘purposive behavior that is based on intentions, integrated into the life course, and modified when unexpected developments occur’ [56] (p.799), so it follows that a close study of the link between life-course events and fertility intentions is important. As Lee [57] has observed, fertility preferences may be ‘moving targets’, therefore questioning whether ‘fertility is an expression of a varying, but temporarily fixed number of desired family size, or whether individuals make their reproductive decisions child by child without having a blueprint of a preferred number of children in mind’ (p.6). Indeed, there is much evidence to suggest the importance of considering step-wise reproductive decision-making (e.g. [58]). To translate this into ‘real-life’ parlance, the life changes that occur after having a first birth may well have an instrumental effect upon the decision to have any more children.

Rather than denigrating the ‘ideal family size’ measurement as a heuristic tool for policymakers and forecasts, it might be more useful to think more concretely about fertility ‘intentions’ as perhaps being somewhat reflective of a short-term ‘business-as-usual’ scenario. In the discussion section we return to this theme to consider how further (qualitative) investigation is required in order to identify precisely what policies might be implemented if policymakers wish to see a demographic future different to ‘business-as-usual’.

In countries characterised by very low fertility such as Taiwan, if we assume a *general* desire to bear children, it could be argued that the key variable to understanding possible future directions of fertility by utilising preference information might be to judge the intentions of moving from having one child to having a second (especially given the very few numbers of higher order births, i.e. third and fourth children). Indeed, Choe et al. [34] have identified a clear difference between ‘ideal’ and ‘planned’ fertility in Korea, with the former not falling below two children over the past two decades and the latter being just 1.4, with only 50% of women with one child planning to have another. Another important element is to understand the gendered component. The stereotypical desire for ‘a boy and a girl’ is well documented, but in societies characterised by very low fertility and histories of privilege accorded to boys such as in Taiwan, does the gender of the first child impact upon the intention to have another?

Using a recent national representative survey, this paper explores fertility intentions in Taiwan with a special focus on women at parity one and parity two. This will form the first full-length examination of fertility intentions in Taiwan published in English and one of the few studies of Pacific Asia that reports a micro-level analysis. By doing this we may be able to provide a better ‘barometer’ of attitudes towards childbearing in Taiwan. Finally, through micro-level analysis, we hope to better identify the predictors of intentions that could, in turn, provide useful clues both for projections as well as shaping policy responses.

## Materials and Methods

The Women’s Marriage, Fertility and Employment Survey is designed to collect information concerning marriage, childbirth status, family composition and job participation. The survey was conducted annually from 1979 to 1988 as a supplement to the Manpower Survey. Since then, however, the survey has been performed at irregular intervals (usually every three to four years). Eligible interviewees are civilian women holding citizenship aged from 18 years. Women in the military and the prison population are excluded. A stratified sampling mechanism is employed and face-to-face interviews are performed by interviewers recruited by *hsien*- (prefecture) or city-level offices of the Directorate General of Budget, Accounting and Statistics.

In early rounds of the survey (e.g. 2000 and 2003) the questions relating to fertility preferences are somewhat different, focussing on ‘ideal number of children’. The questions asked were phrased as follows: ‘How many children do you think is the most ideal to have?’, ‘How many boys do you think is the most ideal to have?’ and ‘How many girls do you think is the most ideal to have?’ In contrast, in the 2006 and 2010 rounds, the respective questions related to the ‘intended number of children’ and were phrased as ‘How many boy births do you expect to give in the future?’ and ‘How many girl births do you expect to give in the future?’ Given our primary focus on fertility *intentions* in this paper, we focus only on these two latter rounds of the survey. These questions were asked only among married and cohabiting women; in the 2006 survey 16,351 women were eligible to answer whereas in the 2010 round there were 15,828 eligible women. Of these, however, only women below age 45 were retained in the analysis, as older women have usually completed their reproductive lives and ‘intended number of children’ has no meaning. This requirement on the data reduced the respective sample sizes to 6,791 women aged 18–44 from the 2006 round and to 5,833 women in this age group from the 2010 round. During this period, fertility continued to fall (albeit at a slower pace) and investment in family policy instruments (which were broadly pro-natalist in their nature) continued to grow [2]. In short, there are few reasons to expect any major change in fertility preferences as derived from an external source (e.g. policy change) between these two surveys.

The descriptive analysis shows percentages of parity zero, parity one and parity two women intending to have further births in 2006 and 2010; the statistical significance of changes in this period is assessed on the basis of the *z*-test. Logistic regression models were used to assess the importance of socio-economic factors and of the gender of the first child among women of parity one who intended to cease childbearing. Socio-economic status is represented by a woman’s educational attainment, her employment status, the husband’s/partner’s educational attainment and whether he is currently unemployed. Education is considered in three groups: the first category includes all women who have completed at the most junior high school (reference category); the second category includes those who have completed senior high school, vocational school and junior college while the third category includes those who have completed at least tertiary education. Partners’ educational attainment was categorised in the same manner. Women’s employment is based on a question about ‘what they were doing’ most of the week before the interview. The main answers were ‘undertaking some kind of work’ or ‘having a job but not at work’ (64.16% N = 905) and ‘housekeeping’ (32.5% N = 459). Hence, the respective variable was divided into three categories, comparing employed and all non-employed (unemployed, retired, long-term ill, etc.) women to housewives. Finally, a variable on the sex of the first child is included, to evaluate the possible effect of gender preference on the decision of a woman to cease childbearing. The models control for number of years since parity one women had their last birth, their age and the age of their husbands/partners. The analysis was performed using STATA 13. The goodness of fit of the models was assessed based on the Hosmer-Lemeshow test.

The data were anonymous when obtained from the Taiwanese government. Ethics approval for this project was obtained from the University of Oxford Central University Research Ethics Committee.

## Results

### Descriptive Findings

As Table 1 demonstrates, a small majority of women at parity zero state an ‘intended’ number of children of two, although this declines slightly between 2006 (60.81%, N = 332) and 2010 (55.06%, N = 310). Intentions to have one child account for 15.38% (N = 84) in 2006, rising modestly to 19.36% (N = 109) in 2010. 22.16% (N = 121) state an intention to remain childless in 2006, rising very slightly to 24.33% (N = 137) by 2010. Nevertheless, none of these abovementioned differences observed between 2006 and 2010 are statistically significant. Overall, the mean intended number of children among childless women decreased slightly in this period from 1.423 (st. dev. 0.86) in 2006 to 1.337 (st. dev. 0.87) in 2010.

**Table 1:** Percentages of women at parity zero by ‘intended’ number of children, 2006 and 2010.

‘Intended’ number of children	2006	2010
0	22.16	24.33
1	15.38	19.36
2	60.81	55.06
3+	1.65	1.24
Sample Size	546	563

With regard to the sub-group of women who intend to remain childless, we note that they have a mean age of 37 in both surveys, and that the most popular *single* reason given for their intentions is ‘health problem or spouse’s health problem’ (2006 35.54%, N = 43; 2010 27.01%, N = 37, detailed tables not shown here). If, however, we consider some of the remaining ‘primary reasons’ for intending childlessness as being related in a group that might be termed ‘socio-economic’–namely ‘financial concerns’, ‘heavy responsibility in raising children’, ‘unwilling to change current lifestyle’–together these account for 48.71% (N = 59) in 2006 and 43.9% (N = 52) in 2010. (NB these ‘reasons’ were only asked for women at parity zero.)

For women at parity one, 54.56% (N = 777) in the 2006 survey and 53.40% (N = 782) in the 2010 survey state an intention to cease childbearing (see Table 2). In 2006, 41.92% (N = 597) state an intention to have one more child while just 3.23% (N = 46) intend to have two further children and 0.28% (N = 4) intend to have at least a further three. In the 2010 survey, these figures remain relatively constant at 40.23% (N = 568) intending one further birth, 5.95% (N = 84) intending two further children and 0.42% (N = 6) intending three or more. In total, the mean intended number of further children of parity one women in 2006 was 0.492 (st. dev. 0.58) compared to 0.534 (st. dev. 0.63) in 2010. Again, most of these slight differences observed over 2006–2010 are not statistically significant, with the exception of the increase in the proportion of women intending to have two more children (*z*-score 3.465, significant at the 1% level).

**Table 2:** Percentages of women at parity one by gender of first birth and ‘intended’ number of further children, 2006 and 2010.

‘Intended’ number of further children	2006			2010		
	1^st^ birth boy	1^st^ birth girl	All	1^st^ birth boy	1^st^ birth girl	All
No more children	60.57	47.38	54.56	57.03	48.89	53.40
One more	35.95	49.07	41.92	36.57	44.76	40.23
Two more	3.22	3.24	3.23	6.39	5.40	5.95
Three or more	0.26	0.31	0.28	0.01	0.95	0.42
Sample Size	776	648	1,424	782	630	1,412

Earlier, we noted the potential role of the gender of the first child in terms of shaping reproductive intentions. At the descriptive level, women with one boy appear more likely to intend to cease childbearing (2006 60.57%, N = 470; 2010 57.03%, N = 446) than if their first child was a girl (2006 47.38%, N = 318; 2010 48.89%, N = 307), with matching relationships in terms of intending to have another child. The difference in intentions between mothers of boys and girls is statistically significant at the 1% level in both rounds of the survey, regarding both ceasing childbearing once they have had a boy (2006 *z*-score 5.00; 2010 *z*-score 3.05) and continuing childbearing if their first child was a girl (2006 *z*-score 5.02; 2010 *z*-score 3.12).

In a society, such as Taiwan, where marriage is a prerequisite for childbearing but women enter in unions rather late and a high proportion of them remains single, progression from the second to the third birth may play an important part in raising fertility rates. As Table 3 shows, the overwhelming majority of women at parity two intend to cease childbearing in both the 2006 (96.47%, N = 3,004) and the 2010 (95.74%, N = 2,654) surveys. Further, no significant difference in the intentions of parity two women over 2006–2010 is observed. Comparing the characteristics of parity two women who intent to continue childbearing to those who do not, their greatest difference is whether they already have had a boy or not. More specifically, 59% of women intending to have at least a third birth in 2006 do not have a boy compared to only 16% of women who intend to cease childbearing; the respective proportions for 2010 are 47% against 17%. Hence, progression to the third birth appears to be driven by son preference.

**Table 3:** Percentages of women at parity two by ‘intended’ number of further children, 2006 and 2010.

‘Intended’ number of further children	2006	2010
No more children	96.47	95.74
One more	3.21	3.97
Two more	0.32	0.29
Sample Size	3,114	2,772

Finally, the data from Tables 1, 2 and 3 suggest that despite continued growth in family policy instruments little direct, immediate impact was reflected in reported fertility intentions.

### Regression Analysis

Table 4 shows descriptive statistics for the variables used in the regression models. The mean age of parity one women aged 18–44 in the 2006 survey is 32.9 years, a year younger compared to the 2010 round. Their husbands are on average age 33.5 in the 2006 round but half a year older in the 2010 round. Mean number of years since their first (and last) birth are very similar for both periods, ranging from 5.7 to 6.0. 54.5% of these women report having a boy as a first child in 2006 whereas the remaining 45.5% have a girl; the corresponding SRB is fairly elevated (1.198). Similarly, in the 2010 round the proportions having a boy are 55.4% compared to 44.6% having a girl; the respective SRB is even higher, 1.244. These parity one women and their husbands are fairly well educated; around one-fifth of them in the 2006 survey have completed at least tertiary education while that proportion reaches one-fourth in the 2010 round. Moreover, as already mentioned there is a high correlation between women’s and men’s educational attainment. The majority of women report themselves as employed in both rounds whereas only about a third corresponds to housewives. A small percentage of husbands are unemployed, 7.4% in the 2006 survey and 14.5% in the 2010 survey.

**Table 4:** Descriptive statistics of the variables in the analysis, women parity one 2006 and 2010 surveys.

Variables		2006	2010
**Demographic variables**			
Woman’s age		32.9 (5.9)	33.8 (5.5)
Husband’s age		33.5 (11.4)	34.0 (12.5)
Years since first birth		5.69 (5.23)	5.97 (5.04)
First birth			
	Girl	45.5	44.6
	Boy	54.5	55.4
**Socio-economic status**			
Woman’s educational attainment			
	Up to junior high school	14.5	11.6
	Up to junior college	65.2	61.5
	At least tertiary	20.3	26.9
Woman’s employment status			
	Housewife	31.2	32.5
	Employed	65.4	61.8
	Unemployed	3.4	5.7
Partner’s educational attainment			
	Up to junior high school	23.4	21.2
	Up to junior college	55.7	51.0
	At least tertiary	20.9	27.8
Partner’s employment status			
	Employed	92.6	85.5
	Unemployed	7.4	14.5
N		1,424	1,412

At the micro-level, after controlling for age of the women, years since first birth and age of their husbands/partners (Table 5), we see a statistically significant greater intention to cease childbearing if the first child is a boy (2006 O.R. 1.583, p>0.000; 2010 O.R. 1.281, p>0.047) and in circumstances where the husband is unemployed (2006 O.R. 2.171, p>0.001; 2010 O.R. 1.236, p>0.036). Conversely, the intention to cease childbearing is lower among women whose partner has completed secondary education (2006 O.R 0.673, p>0.043; 2010 O.R 0837, p>0.399) or tertiary qualifications (2006 O.R. 0.502, p>0.007; 2010 O.R. 0.791, p>0.350), though this is not a significant result regarding 2010. Educational attainment of the mother or her employment status are not significant predictors.

**Table 5:** Predictors of ceasing childbearing among women of parity one 2006 and 2010.

Predictors		2006	2010
		OR (95% CI)	OR (95% CI)
**Demographic variables**			
Woman’s age		1.104 (1. 070, 1.140)	1.120 (1.084, 1.157)
Husband’s age		1.020 (1.005, 1.035)	1.011 (0.998, 1.026)
Years since first birth		1.202 (1.151, 1.256)	1.150 (1.07, 1.194)
First birth			
	Girl (ref. cat.)	1.000	1.000
	Boy	1.583 (1.231, 2.035)	1.281 (1.003, 1.637)
**Socio-economic status**			
Woman’s educational attainment			
	Up to junior high school (ref. cat.)	1.000	1.000
	Up to junior college	1.218 (0.922, 1.610)	0.775 (0.482, 1.246)
	At least tertiary	1.307 (0.631, 2.708)	0.757 (0.433, 1.322)
Woman’s employment status			
	Housewife (ref. cat.)	1.000	1.000
	Employed	1.218 (0.922, 1.610)	1.112 (0.849, 1.455)
	Unemployed	1. 307 (0. 631, 2.708)	0.962 (0.546, 1.694)
Partner’s educational attainment			
	Up to junior high school	1.000	1.000
	Up to junior college	0.673 (0.459, 0.987)	0.838 (0.555, 1.264)
	At least tertiary	0.502 (0.306, 0.826)	0.791 (0.484, 1.293)
Partner’s employment status			
	Employed (ref. cat.)	1.000	1.000
	Unemployed	2.171 (1.291, 3.650)	1.236 (1.014, 1.508)
Pseudo R^2^		24.97	20.89

## Discussion and Conclusions

Before discussing the findings presented above, we can first reflect on some of the limitations of this study. Firstly, we are limited by the questions in the survey and are therefore unable to explore some potentially interesting avenues relating perhaps to types of employment, family-friendly environments, gender attitudes, childcare arrangements, costs of living and so on. We return to this at the end of this section. Secondly, because of the change of questions in the mid-2000s we are only able to consider two rounds of the survey taking place only four years apart. However, the relative consistency of the two surveys as presented in the results section above (and the fact that they both herald from eras of equally low fertility) suggests that it is unlikely that either dataset is an outlier; hence, they serve to reinforce conclusions but not to observe changes or trends overtime. A third limitation relates to the very nature of the survey. In common with other Pacific Asian surveys on fertility intentions, the survey under analysis here only covers married women. This retards direct comparisons with European and North American surveys, which tend to cover both married and unmarried women. Furthermore, in the context of a ‘retreat from marriage’ as identified above and much later marriage coupled with only a few instances of birth outside of marriage, it lends us very little information about the increasing number of men and women who remain single well into their 30s –a phenomenon that plays a large role in driving very low fertility.

A number of observations can be made at the descriptive level. First, the evidence of ‘intentions’ of those with no children do, indeed, lend credence to the notion of a two-child norm *à la* Sobotka and Beaujouan [30] as two is the most popular number of intended children (even if the mean is much lower). However, given our previous discussion concerning the questionable existence of a ‘blueprint’ of preferred number of children, these figures might be better interpreted again as ‘ideals’ rather than necessarily as definitive ‘intentions’. For the couples who state an intention to remain childless, the evidence appears to suggest a combination of biological and socio-economic factors at play. Yet, given the porous relationship between definitions of ‘voluntary’ and ‘involuntary’ childlessness [59], the possible role of subfecundity in shaping the reported intentions of those who cite these ‘other’ reasons should not be completely disregarded. In other words, this may not be a ‘binary’ representation of reality. Clearly further (possibly qualitative) research is required to disentangle these reasons for intended childlessness in order to gauge the possible strength of the role of self-actualisation (and its relationship with individualism and consumption preferences) associated with theories of the ‘second demographic transition’ [25].

The ‘intentions’ of women at parity one appear to map onto the expected reality of childbearing in Taiwan when judging from the actual fertility rates presented above. As intimated earlier, this could lead to the suggestion that the ‘business as usual’ scenario (i.e. net of any significant policy intervention) might be a continuance of very low fertility if, indeed, these intentions are realised. Furthermore, these findings are very similar to those reported by Choe et al. [34] for South Korea (see above). Clearly, there is some disparity between the ‘ideal’ number of two children regularly presented as a ‘norm’ in Taiwan and these descriptive measurements of ‘intentions’.

Given Taiwan’s recent history of son preference [60], it is perhaps unsurprising that there is some bias towards ceasing childbearing with one son over one daughter. At the descriptive level these effects appear relatively modest though statistically significant, in accordance with the general trend towards ‘gender indifference’ regarding children since 1990, as reported by Lin [61]. However, the regression analysis indicates an effect of the gender of the first-born child upon future fertility intentions. In an era of easy access to sex-selective technology, however, it is difficult to disentangle these findings in order to determine the extent to which these reflect the actualisation of a ‘blueprint’ of a family by gender and parity, or otherwise. Apart from the importance of a second birth in rising fertility rates, having a third birth can be a decisive factor pushing fertility up to approach replacement level, especially in a society like Taiwan which is characterised by late age at marriage and high proportions of single persons. The analysis indicates that the lack of a son determines to a large extent the decision of a woman to have a third child.

Turning to the regression analysis, we identify the important role of husbands’ unemployment in curtailing intentions to have further children. This concurs with the broader literature on this relationship (see reviews in [62,63]). In providing only a generally binary variable of male and female employment, it is not possible to examine the potential role of fragile or flexible employment on fertility intentions–indeed a prior study has suggested that female self-employment, similar to past experiences of informal labour discussed above, may lead to easier work-family reconciliation in Taiwan [64].

In the classic ‘Home Economics’ model of childbearing, highly educated women substitute ‘quantity’ for ‘quality’ in order to mitigate the higher opportunity costs of childbearing and childrearing [65,66]. For men, meanwhile, who generally spend less time with children, the positive income (or education) effect tends to dominate [67].

However, this model is being increasingly challenged by a number of recent studies of European women [68–71]. The author of one such study concluded that ‘in institutional contexts allowing highly educated women to have large families, women of reproductive ages are more prone to make investments in both human capital and family size, because these choices are not seen as incompatible alternatives’ (p.28). In particular, Becker [72] argues that the higher incomes of educated women when coupled with their partners (usually higher educated, higher income) may better position them to absorb the costs of a larger family. Finally, as Mills et al. [71] observe for the European context, there is a tendency for more highly educated men and women to be matched in more gender equal partnerships where the male partner makes a greater contribution to home and childcare duties.

Our regression exercise did not, however, find a statistically significant relationship between female education and fertility intentions. Nevertheless, the exercise did suggest that increasing levels of the husband’s education were negatively related to an intention to cease childbearing at parity one. Numerous studies have identified a general shift towards more progressive attitudes among (especially young) men in Taiwan regarding childcare, housework and more general notions of ‘fatherhood’ [73,74]. As marriages become ever more equal and homogenous between partners with equally high education, there is some potential for fertility intentions to be increased in the future as a result of this process [75].

Without an improved understanding of the drivers behind these stated intentions it is not possible to adequately define a ‘prescription’ of policy responses. This would require both more targeted, deeper survey instruments as well as in-depth qualitative studies. Only by doing so will we get a better understanding (beyond a macro-sociological view) of the reasons *why* so many women in Taiwan *intend* to stop childbearing at parity one and what makes them so different (in this regard) to the majority of women in Europe. For example, an in-depth qualitative investigation of the experiences of new parents in Taipei is currently under way [76]. This attempts to explore the ways in which having a first child impacted upon various aspects of life; how these were aligned to expectations; and how this experience shaped their views towards having a second child. The preliminary findings identify a complex web of issues relating not only to areas which might be amenable to policy interventions (such as economic pressures and so on) but also to more psychological and physical issues relating to ‘me time’ and ‘bodily strength’, with the latter especially important for mothers giving birth at later ages.

The employment of the notion of a ‘two-child norm’ has the potential to present a misleading impression of the culture of childbearing in Taiwan. In terms of presenting a ‘positive’ space for policymakers to ‘fill’ with simple pro-natalist measures, it could be argued that the notion of a ‘two-child norm’ as represented by *ideal* families ‘flatters to deceive’. Rather, from the evidence in this survey Taiwanese women appear to have largely reconciled their childbearing trajectories to their circumstances with a majority of women with one child intending to cease childbearing. Again, though, this is *under current circumstances*, and ‘unforeseen developments’ either at the level of policy, or within the household, could still change these preferences and plans. Finally, while the ‘low fertility trap hypothesis’ considers personal ideal family sizes, the extent to which sub-replacement *intentions* become a long-term feature of a society could be a better reflection of the degree to which small family sizes have become ‘normalised’ and, therefore, make (implicit or explicit) fertility raising policies more difficult to succeed with.

Only by moving beyond these narrow (and false) conceptions of ‘filling the gap between *ideal* and actual fertility’ and developing a better understanding of the malleability of these *intentions* (i.e. are they fixed life plans or are they pragmatic responses to circumstances?) can we begin to design policies that allow men and women to have the number of children that *they would like*, rather than that *the state would like*. Finally, while studying the fertility preferences of the unmarried might be open to criticisms regarding being (possibly unrealistic) ‘ideals’, they are simply too large a constituency to overlook. By fostering a more inclusive survey methodology, and exploring in greater depth the reasons for staying single, we would be able to come close to completing the picture of the reproductive horizons of Taiwanese men and women.
